# Autonomous driving controllers with neuromorphic spiking neural networks

**DOI:** 10.3389/fnbot.2023.1234962

**Published:** 2023-08-11

**Authors:** Raz Halaly, Elishai Ezra Tsur

**Affiliations:** Neuro-Biomorphic Engineering Lab, Department of Mathematics and Computer Science, Open University of Israel, Ra'anana, Israel

**Keywords:** autonomous driving, neuromorphic control, spiking neural networks, path-tracking controllers, neural engineering framework (NEF), energy efficiency, motion planning, computational frameworks

## Abstract

Autonomous driving is one of the hallmarks of artificial intelligence. Neuromorphic (brain-inspired) control is posed to significantly contribute to autonomous behavior by leveraging spiking neural networks-based energy-efficient computational frameworks. In this work, we have explored neuromorphic implementations of four prominent controllers for autonomous driving: pure-pursuit, Stanley, PID, and MPC, using a physics-aware simulation framework. We extensively evaluated these models with various intrinsic parameters and compared their performance with conventional CPU-based implementations. While being neural approximations, we show that neuromorphic models can perform competitively with their conventional counterparts. We provide guidelines for building neuromorphic architectures for control and describe the importance of their underlying tuning parameters and neuronal resources. Our results show that most models would converge to their optimal performances with merely 100–1,000 neurons. They also highlight the importance of hybrid conventional and neuromorphic designs, as was suggested here with the MPC controller. This study also highlights the limitations of neuromorphic implementations, particularly at higher (> 15 m/s) speeds where they tend to degrade faster than in conventional designs.

## 1. Introduction

Path and motion planning are crucial aspects of autonomous driving systems (ADSs) (Huang et al., 2019b). ADSs encompass a wide range of approaches, ranging from classic control theory (Alcala et al., 2018) to machine learning (Qureshi et al., 2019). Typically, ADSs consist of three main components: environment sensing and localization (Ji et al., 2019), path planning (Artuñedo et al., 2018), and path tracking (Sun et al., 2019). ADSs are realized using problem formulation (Arkin, 1990), and optimization criteria (Liu et al., 2022, 2023a) (such as vehicle stability Li et al., 2009 and safety Huang et al., 2019a), and typically require significant computational and energy resources (Gawron et al., 2018).

Neuromorphic brain-inspired control systems, which are based on densely connected spiking neural networks (SNNs) (Tsur, 2021), offer a promising alternative with greater energy efficiency and comparable accuracy and latency (DeWolf et al., 2020), DeWolf (2021) to system control. In this work, we propose a neuromorphic implementation of four well-established path-tracking control models for autonomous driving (Samak et al., 2021), within a physics-aware computational framework. Our proposed ADSs utilize a LiDAR sensor to estimate the vehicle's position along the track. We used LiDAR readings to generate a reference trajectory and employs neuromorphic approaches for path tracking through the implementation of the following controllers: 1. Pure-pursuit (Coulter, 1992)—A widely adopted path-tracking controller (Morales et al., 2009), which geometrically pursues a point on the reference trajectory by adjusting the vehicle's steering angle; 2. Stanley (Hoffmann et al., 2007) controller, a controller that was instrumental in the first-place victory of the 2005 DARPA Grand Challenge for autonomous driving (Hoffmann et al., 2007). It adjusts the vehicle's steering to minimize both cross-track error (CTE; defined as the distance between the front axle of the vehicle to the closest point on the reference (or ideal) path) and heading error (defined as the angle between the vehicle and the trajectory headings); 3. PID controller, a commonly used controller that continuously decreases the CTE by applying corrections based on the error's proportional, integral, and derivative terms; and 4. Model predictive control (MPC), which employs a predictive model to evaluate the system's future state and optimizes the control policy accordingly. In contrast to conventional artificial neural networks-based controllers, which optimize long-term policies but may pose unexplained, unsafe, and harmful consequences in the short term (Alcala et al., 2018), we chose these control models as they are widely utilized in reliable strategies (Samak et al., 2021) and offer comfortable, safe, explainable, and interpretable motion control (Berkenkamp et al., 2017).

We employed the neural engineering framework (NEF), a widely adopted neuromorphic computing framework, for designing our neuromorphic implementations (Eliasmith and Anderson, 2003; Tsur, 2021). NEF provides mathematical constructs that enable encoding, decoding, and transformation of numerical values using spiking neurons, facilitating the implementation of functional large-scale SNNs (Eliasmith and Anderson, 2003). NEF has been used in the design of various neuromorphic systems spanning robotics control (DeWolf et al., 2020) and visual processing (Tsur and Rivlin-Etzion, 2020) to perception (Cohen Duwek and Ezra Tsur, 2021; Cohen-Duwek et al., 2022). Additionally, the framework has been demonstrated on prominent digital neuromorphic hardware architectures, including TrueNorth (Fischl et al., 2018), the Loihi (Lin et al., 2018), the NeuroGrid (Boahen, 2017), and the SpiNNaker (Mundy et al., 2015), as well as deployed on dedicated analog circuitry (Hazan and Ezra Tsur, 2022). We utilized Nengo (Bekolay et al., 2014), a Python-based neural compiler built on NEF principles, to translate high-level functional descriptions to low-level neural models (Bekolay et al., 2014).

This work aims to assess the viability of neural approximation methods for controlling ADSs and offer guidelines for designing neuromorphic control strategies.

## 2. Methods

**Figure 1:**
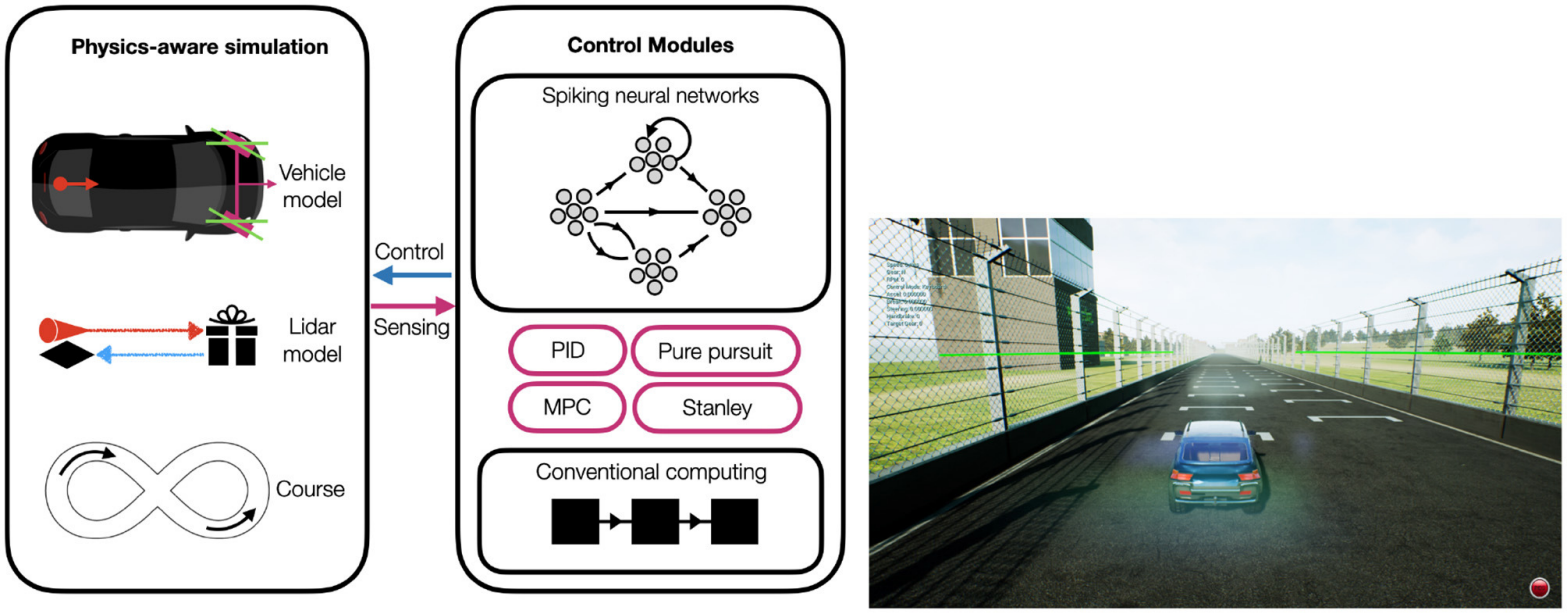
Schematic system design. The AirSim's SUV inside the FST Driverless Environment, senses the environment with LiDAR (The green lines). The control module computes the desired control signal.

In this section, we present NEF, which serves as the theoretical foundation of our neuromorphic designs, and the kinematic bicycle model (KBM) with which we modeled the vehicle. We also describe the path-tracking controllers and the simulation environment we employed in this study. Generally, the goal of the path tracking controllers is to enable an autonomous vehicle to follow a reference trajectory while minimizing errors and maintaining desired performance; and the simulation environment serves as a platform for testing and comparing the performance of the different controllers (Figure 1).

### 2.1. Neural engineering framework

The NEF defines three principles for designing neuromorphic spiking neural networks: representation, transformation, and dynamics. It is described in length in Eliasmith and Anderson (2003) and Tsur (2021).

#### 2.1.1. Principle 1 - representation

An ensemble of neurons represents a time-varying vector of real numbers through nonlinear encoding and linear decoding. The encoding is responsible for representing numerical constructs as spikes. The encoding of an input vector *x* is defined by:


(1) $$\begin{array}{l} \delta_{i}\left(x\right)=G_{i}\left[\alpha_{i}\langle {\tilde{\phi }}_{i},x\left(t\right)\rangle +J_{i}^{bias}\right], \end{array}$$

where *G*_*i*_ is a nonlinear function that represents a neuron model (here, we use the leaky-integrate-and-fire (LIF) (Burkitt, 2006) model), α is a gain term, $\tilde{\phi }$ is the neuron's preferred stimulus (the value for which the neuron is firing at the highest rate), and *J*_*i*_ is a bias fixed current. The estimated represented vector $\hat{x}$ can be decoded using a linear decoder:


(2) $$\begin{array}{l} \hat{x}\left(t\right)=\Sigma_{i}\left(h_{i}\left(t\right)*\delta_{i}\left(t\right)\right)d_{i}^{x}, \end{array}$$

where *d*_*i*_ are linear decoders that were optimized to reproduce *x* by using least squared optimization, and *h*_*i*_ is a filter that convolved with δ_*i*_ for representing the spiking activity.

#### 2.1.2. Principle 2 - transformation

The decoders *d*_*i*_ can be optimized to reproduce any *f*(*x*) using least squared optimization (Eliasmith and Anderson, 2003). Similarly to (2), $\hat{f}\left(x\right)$ can be decoded using:


(3) $$\begin{array}{l} \hat{f}\left(x\left(t\right)\right)=\Sigma_{i}\left(h_{i}\left(t\right)*\delta_{i}\left(t\right)\right)d_{i}^{f}. \end{array}$$

*f*(*x*) can be calculated using a set of weighted synaptic connection *w*_*ij*_, connecting two neural ensembles *A* and *B*:


(4) $$\begin{array}{l} f\left(x\right)=w_{ij}=d_{i}\cdot e_{i}, \end{array}$$

where *d*_*i*_ are the decoders of ensemble *A* and *e*_*j*_ are the encoders of ensemble *B*. This allows the neuromorphic approximation of any function in a SNN.

#### 2.1.3. Principle 3 - dynamics

A canonical form of a linear error-correcting feedback loop can be described using:


(5) $$\begin{array}{l} ẋ=Ax\left(t\right)+Bu\left(t\right), \end{array}$$

where *A* is a dynamics matrix and *B* is the input matrix, *x* is the state vector, and *u* is the input vector.

This standard control can be realized in NEF by using:


(6) $$\begin{array}{l} ẋ=A^{\prime }x\left(t\right)+B^{\prime }u\left(t\right), \end{array}$$

where *A*′ is a recurrent connection and *B*′ is the input connection. These matrices can be related to the standard dynamics and input matrix using *A*′ = τ*A*+*I* and *B*′ = τ*B* (Eliasmith and Anderson, 2003).

Such a recurrent connection is defined, by the previous principles, using


(7) $$\begin{array}{l} x\left(t\right)=f\left(x\left(t\right)\right)*h\left(t\right). \end{array}$$

This neural implementation can also be used to implement a neuromorphic integrator. An integrator that integrates *u* to define *x* where ẋ = *u*, can be defined as *A* = 0, *B* = 1 in terms of (5) and *A*′ = 1, *B* = τ in a neuromorphic system with a recurrent connection in terms of (6). The utilization of NEF's dynamics for data integration is further described in detail in Tsur (2021) and Zaidel et al. (2021).

### 2.2. The kinematic bicycle model

We used the KBM to model the steering of our 4 wheels car. The KBM is a simplified vehicle model commonly used in control and robotics applications to describe the motion of a vehicle. It approximates the vehicle as a rigid body with two wheels, one at the front and one at the rear, connected by a fixed wheelbase. KBM represents the vehicle's state as [*x, y*, θ, δ], where *x* and *y* represent the vehicle's position, θ is the vehicle's heading angle, and δ is the vehicle's steering angle. The model's input is [*v*, φ], where *v* is the vehicle's velocity, and φ is its steering rate. The vehicle's position was represented in two-dimensional space in reference to one of the following points: the center of the front axle, the center of the rear axle, and the vehicle's center of gravity. When using the center of the rear axle as a reference point, the new state of the vehicle is obtained by:


(8) $$\begin{array}{l} \begin{array}{c} x\left(t+1\right)=x\left(t\right)+v\cos\left(\theta \left(t\right)\right)\Delta t,\\ y\left(t+1\right)=y\left(t\right)+v\sin\left(\theta \left(t\right)\right)\Delta t,\\ \theta \left(t+1\right)=\theta \left(t\right)+v\tan\delta \left(t\right)\Delta t,\\ \delta \left(t\right)=\delta \left(t\right)+\varphi \Delta t. \end{array} \end{array}$$

When using the center of the front axle as a reference point, the new state of the vehicle is:


(9) $$\begin{array}{l} \begin{array}{c} x\left(t+1\right)=x\left(t\right)+v\cos\left(\theta \left(t\right)+\delta \left(t\right)\right)\Delta t,\\ y\left(t+1\right)=y\left(t\right)+v\sin\left(\theta \left(t\right)+\delta \left(t\right)\right)\Delta t,\\ \theta \left(t+1\right)=\theta \left(t\right)+v\sin\delta \left(t\right)\Delta t,\\ \delta \left(t\right)=\delta \left(t\right)+\varphi \Delta t. \end{array} \end{array}$$

A detailed description, as well as a description of the model's advantages and limitations, are described in Polack et al. (2017).

### 2.3. Path tracking controllers

Path tracking controllers are essential components of autonomous vehicle systems, responsible for generating steering and throttle commands to follow a reference trajectory. In this study, we investigate four different controllers: Pure-pursuit, Stanley, PID, and MPC. Each controller features a unique architecture, with its own unique set of parameters and performance. We provide below a brief introduction to each controller, followed by a description of their neuromorphic implementations.

#### 2.3.1. PID controller

A PID controller is widely utilized in systems that require continuous error-correcting control. A PID controller reduces an error *e*(*t*) by providing a control signal *u*(*t*) that applies the required corrections based on the error's proportional, integral, and derivative terms, using:


(10) $$\begin{array}{l} u\left(t\right)=K_{p}e\left(t\right)+K_{i}\overset{t}{\underset{0}{\int }}e\left(t\right)dt+K_{d}\frac{de}{dt}, \end{array}$$

where *K*_*p*_, *K*_*i*_, and *K*_*d*_ are the proportional, integral, and derivative gain coefficients, respectively.

Our neuromorphic PID implementation was described in length in Shalumov et al. (2021). In short, The vehicle's current configuration (e.g., speed) was introduced through a neuron ensemble. We subtracted it from the desired configuration signal (e.g., target speed) to derive an error signal. This error signal was propagated to the output ensemble through three paths: 1. A proportional path in which the error is multiplied through a gain factor; 2. An integration path in which the error is integrated using a neuromorphic integrator (see Section 2.1.3) and scaled by a gain factor; and 3. A derivative path, implemented by connecting the error ensemble to a 2D derivative ensemble. To implement derivation, the error was propagated through slow and fast synapses which were sequentially subtracted and scaled by a gain factor. These three error signals were summed in an output ensemble and delivered to the vehicle.

**Figure 2:**
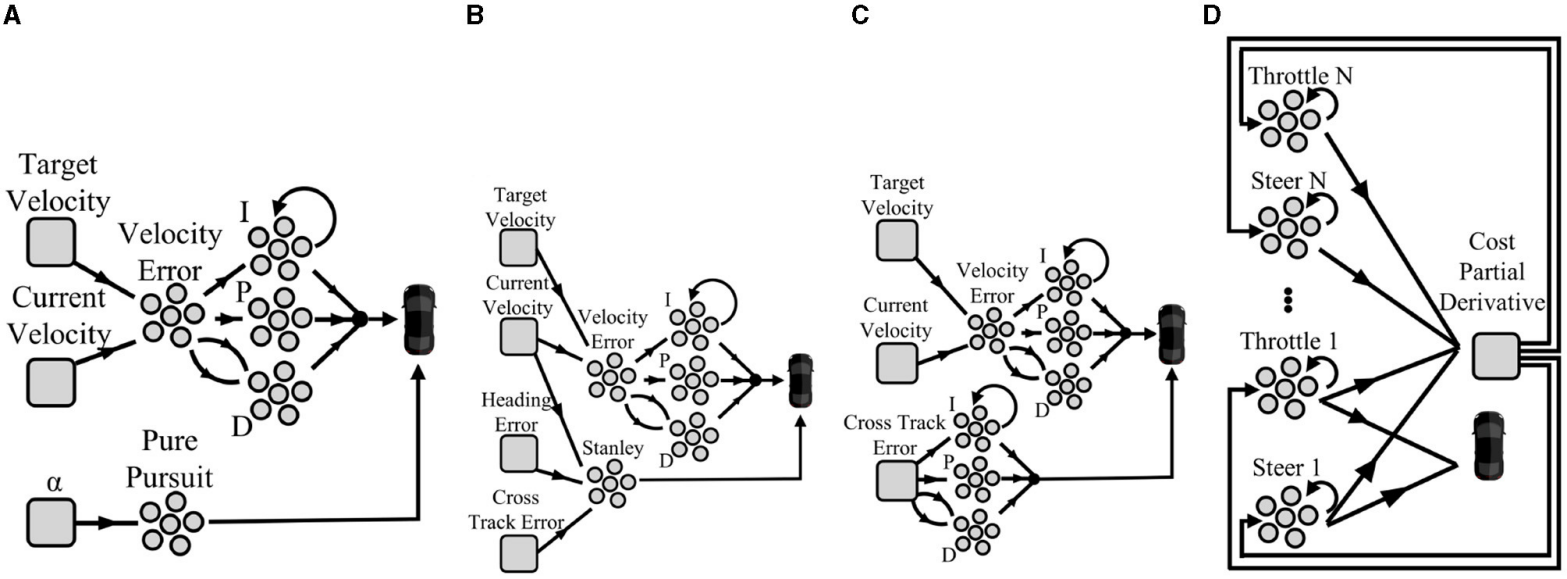
Schematic design of the controllers implemented as an SNN. **(A)** Pure-pursuit, **(B)** Stanley, **(C)** PID, **(D)** MPC.

Our vehicle's PID controller was implemented by using two PID controllers. One PID was used as a cruise control, responsible for throttle adjustment and the second one was used for steering. For controlling the steering, the error signal was calculated using:


(11) $$\begin{array}{l} u\left(t\right)=e\left(t\right)+v\left(t\right)\sin\psi \left(t\right), \end{array}$$

where *e*(*t*) is the CTE (the distance between the front axle of the vehicle to the closest point on the reference path), *v*(*t*) is the vehicle's speed, and ψ(*t*) is the vehicle's heading error (the angle between the vehicle and the trajectory headings). The error signal combines the CTE and the heading error in such a way that the PID is used to reduce the error by adjusting the control signal accordingly. The PID model scheme is illustrated in Figure 2C.

As a baseline, we set the steering PID gains to *K*_*p*_ = 0.7, *K*_*i*_ = 0.1, *K*_*d*_ = 0.3, and the synaptic time constants to τ_*p*_ = 5*ms*, τ_*i*_ = 200*ms*, τ_*d*_ = 500*ms*. For the CPU-based implementation, we chose: *K*_*p*_ = 0.2, *K*_*i*_ = 0.01, *K*_*d*_ = 0.3. We evaluated the model with 10, 25, 50, 100, 250, 500, 1000, and 2500 neurons per ensemble. The steering PID synapses (τ_*p*_, τ_*i*_, τ_*d*_) were evaluated with time constants of 5, 10, 50, 100, 300, 500, 700, 900, and 1100 ms. The rest of the synapses were set to have a time constant of 5 ms (low pass filter). All ensembles were configured with a radius of 1.

#### 2.3.2. Pure-pursuit controller

The Pure-pursuit controller is a path-tracking controller, which geometrically chases a point on a reference trajectory some distance ahead of the vehicle. It uses the vehicle's rear axle as the reference point and a constant distant point on the reference trajectory as a target point. Steering angle δ at time *t* is calculated using


(12) $$\begin{array}{l} \delta \left(t\right)=\arctan\left(\frac{2L\sin\alpha }{l_{d}}\right), \end{array}$$

where *l*_*d*_ is the distance between the vehicle's reference point and the target point (look-ahead distance), *L* is the distance between the vehicle axles, and α is the angle between the vehicle's body and the target point.

Our neuromorphic implementation of the pure-pursuit controller was based on one one-dimensional neuronal ensemble that represents α. Its decoders were optimized to calculate the steering angle from Equation (12). The pure-pursuit controller does not control the vehicle's speed. Therefore, to maintain the vehicle's velocity, we utilized a neuromorphic PID-based cruise control as described above. The pure-pursuit model scheme is illustrated in Figure 2A.

We set the PID gains for the CPU implementation a:s *K*_*p*_ = 0.5, *K*_*i*_ = 0.02, *K*_*d*_ = 1, and for the neuromorphic implementation as *K*_*p*_ = 1.3, *K*_*i*_ = 0.9, *K*_*d*_ = 0.5', with the synaptic filters τ_*p*_ = 0.005*s*, τ_*i*_ = 0.2*s*, τ_*d*_ = 0.3*s*. We evaluated the model with 10, 25, 50, 100, 250, 500, 1,000, and 2,500 neurons per neuromorphic ensemble. The output synapses were set as low-pass filters. We evaluated the steering synapses with time constants of 5, 10, 50, 100, 300, 500, 700, 900, and 1,100 ms. The rest of the synapses were set to have a time constant of 5 ms (low pass filter). The neuromorphic ensembles were set with a radius of 1. This representational radius determines the range of the ensembles' acceptable input values. We set the controller's look-ahead to 8 m and set the synaptic filter (τ) of the output synapse connections to 10 ms.

#### 2.3.3. Stanley controller

The Stanley controller (Hoffmann et al., 2007) is a path-tracking controller, which instead of chasing a path, strives to minimize the CTE (*e*(*t*)) and heading (ψ(*t*)) errors through geometrical calculations. The Stanley controller uses the front axle of the vehicle as the reference point. The required steering angle δ(*t*) was calculated using:


(13) $$\begin{array}{l} \delta \left(t\right)=\psi \left(t\right)+\tan^{-1}\left(\frac{ke\left(t\right)}{k_{s}+v\left(t\right)}\right), \end{array}$$

where *k* and *k*_*s*_ are adjustable softening coefficients for the controller. *k* is the error's gain factor, and *k*_*s*_ provides the means for controlling the vehicle at low speeds when the denominator becomes small and the steering becomes too aggressive.

The neuromorphic implementation of the Stanley controller comprised one three-dimensional neuronal ensemble that represents *e*(*t*), ψ(*t*), and *v*(*t*). Its decoders were optimized to calculate the steering using Equation 13. Similarly to the pure-pursuit controller, the Stanley controller does not control the vehicle's speed, which is maintained using neuromorphic PID-based cruise control. The Stanley model scheme is illustrated in Figure 2B.

The Stanley neuromorphic implementation requires a three-dimensional ensemble with a larger radius of $\sqrt{3}$, and as a result, a higher number of neurons. The other 1-dimensional synapses were configured with a radius of 1. We evaluated the model with 100, 250, 500, 1,000, 2,500, 5,000, and 10,000 neurons per neuromorphic ensemble. The model's output steering synapses were set with time constants of 5, 10, 50, 100, 300, 500, 700, 900, and 1,100 ms. The rest of the synapses were set to have a time constant of 5 ms (low pass filters). We set the *k* and *k*_*s*_ softening coefficients to 1.

#### 2.3.4. MPC controller

The Model Predictive Control (MPC) controller is an advanced optimization-based feedback loop control paradigm, which uses a model to predict the state of the system in the future and uses it to find the optimal driving policy for a given goal. MPC can handle multiple inputs and outputs that might interact with each other (e.g., steering and throttle values), increasing the potential for better overall performance. MPC can also handle constraints, allowing it to avoid undesired or impossible scenarios, like increasing throttle values to physically-impossible levels.

The MPC controller derives *N* predictions for a given elapsed time Δ*t*. *N*·Δ*t* represents the controller's prediction horizon, which determines how far the model can look ahead into the future. Although the controller provides *N* prediction, only the first prediction, which represents the next timestamp, is used as the control command. Once that command is executed, the controller will retrieve the new state of the system and provide another set of predictions.

Here, to implement the MPC controller we defined the cost function:


(14) $$\begin{array}{l} \begin{array}{cc} Cost= & 50\Sigma_{k\in N}e_{k}^{2}+100\Sigma_{k\in N}\psi_{k}^{2}+\\ 100\Sigma_{k\in N}{\left(v_{ref}-v_{k}\right)}^{2}+100\Sigma_{k\in N}\delta_{k}^{2}+\\ 1\Sigma_{k\in N}a_{k}^{2}+200\Sigma_{k\in N}{\left(\delta_{k}-\delta_{k-1}\right)}^{2}+\\ 10\Sigma_{k\in N}{\left(a_{k}-a_{k-1}\right)}^{2}, \end{array} \end{array}$$

where *e* is the CTE, ψ is the heading error, *v*_*ref*_ is the target velocity, *v* is the vehicle velocity, and δ and *a* are the chosen steering and throttle policies, respectively. The four last components in Equation 14 reduce the actuators' usage and rate of change. The MPC's prediction horizon comprises the steering and the throttle values for each timestamp, yielding 2*N* parameters to optimize.

To implement the MPC in a CPU, we used the Sequential Least Squares Programming (SLSQP) minimization algorithm as an optimizer, an iterative method for constrained nonlinear optimization (Boggs and Tolle, 1995). Our neuromorphic controller is a hybrid controller that uses an SNN for optimization and a CPU to accurately resolve the required mathematical calculations. The neuromorphic model of the MPC was defined as a closed-loop network, comprising 2*N* neural ensembles that represent the required steering and throttling control signals for *N* predictions. Each ensemble was defined as an integrator with a recurrent synapse, which acts as a memory. All these ensembles were connected through synapses to a CPU block that calculates the cost function. The cost function is resolved by a CPU, applying root mean squared propagation (RMSprop) on the estimated partial derivative of the cost function. RMSProp is a gradient descent-driven algorithm that uses a decaying moving average of partial gradients to adapt the step size of each optimized variable, emphasizing recent partial gradients over early gradients, overcoming the limitation of more conventional implementations (Kurbiel and Khaleghian, 2017). Notably, since the neuromorphic model works continuously without a clock, unlike the CPU implementation, the optimization is done continuously by updating the policies based on the current state without redoing the optimization from scratch at each timestamp.

We configured the RMSprop algorithm with a decay rate of 0.9. The partial derivative of the cost function (Equation 14) was estimated using:


(15) $$\begin{array}{l} \frac{\partial f}{\partial x_{k}}=\frac{f\left(x_{1},...,x_{k}-\varepsilon ,...,x_{n}\right)-f\left(x_{1},...,x_{k},...,x_{n}\right)}{\varepsilon }, \end{array}$$

where ε was set to 0.01.

Once RMSprop was executed, the step values were transferred back to the neuromorphic integrators. Each integrator receives the cost derivative as an input and has a recurrent connection that persists in its spiking dynamic. Each neuromorphic integrator represents a single value and their decoders were optimized to realize an identity function, realizing a memory cell. The integrators values increase and decrease per the continuous realization of the represented cost. The MPC is illustrated in Figure 2D.

We evaluated the model with 10, 25, 50, 100, 250, 500, 1,000, and 2,500 neurons per ensemble. The model's output synapses were set with time constants of 5, 10, 50, 100, 300, 500, 700, 900, and 1,100 ms. The recurrent synapse of each integrator was set with a time constant of 200 ms. The rest of the synapses were set to have a time constant of 5 ms (low pass filter). All neuromorphic ensembles were defined with a radius of 1. Interestingly, the PID's integral ensemble radius prevents the integral windup problem (PID's integral term might accumulate errors and overshoots due to its unwound value).

### 2.4. Simulation Environment

**Figure 3:**
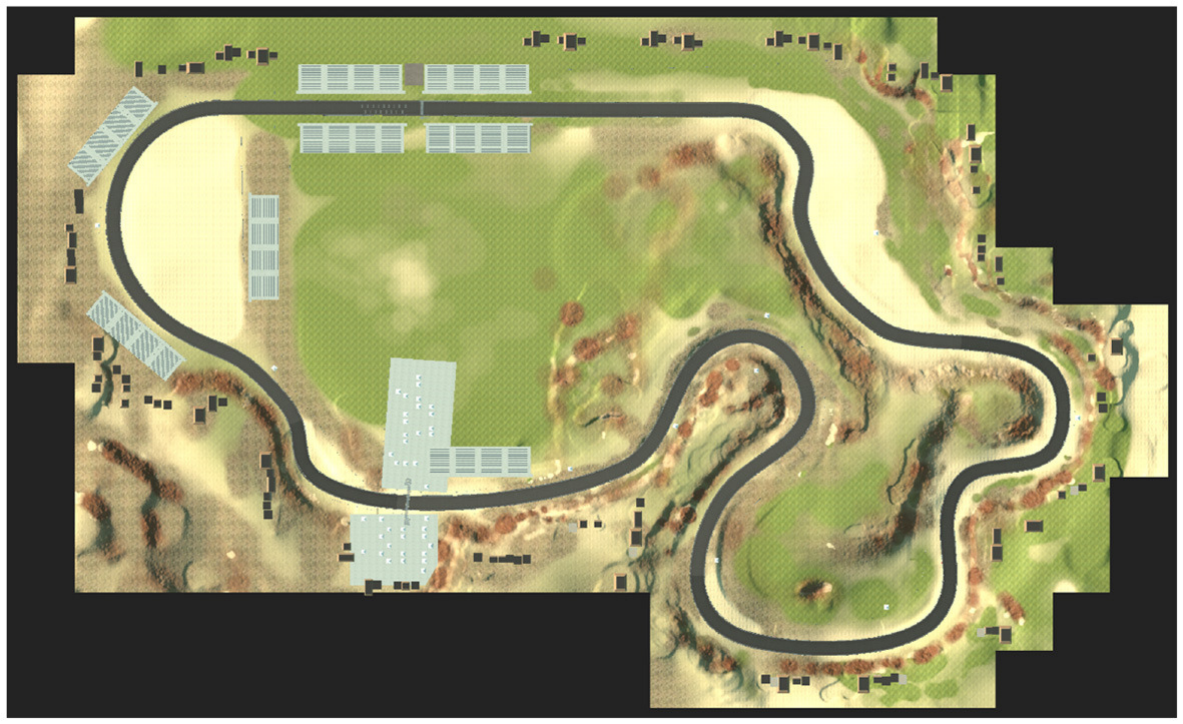
A top view of the FST driverless environment.

We used the AirSim simulator (Shah et al., 2017), a physics-aware Unreal Engine-based, open-sourced, cross-platform framework, which was developed by Microsoft to simulate a controlled vehicle in a realistic racecourse environment. The race course was based on the FST Driverless Environment (Zadok et al., 2019) and we added solid, wall-like sides, lining a 15 meters width road (Figure 3).

We used AirSim's plausible physical model of an SUV vehicle (Shah et al., 2017) and a LiDAR sensor, covering a 180° field of view in a half-degree resolution a 40-meter range, working at 40 scans per second.

The Neuromorphic NEF-based controllers were implemented using the Nengo library (Bekolay et al., 2014), and the conventional CPU-based controllers were implemented using Python. We designed an adapter to allow synchronized communication between the Nengo and the AirSim environments, allowing the exchange of LiDAR readings and control signals (car's steering and throttle values). Scenarios were executed synchronously with a 5-millisecond interval, emulating 200 control signals per second. Driving policies require a reference trajectory to follow. Here, we used the race course's mid-line as a reference. The path was generated in real-time, using the LiDAR readings (detecting the race course's lining walls), and represented using a third-degree polynomial. This polynomial was derived by calculating the center point between the boundaries at evenly spaced intervals. To reduce any non-deterministic effect, we run each experiment 10 times and set a constant seed value for the random mechanism.

Given the intrinsic time-dependent nature of SNNs, we have our framework also had to handle time-space synchronization between the AirSim and Nengo simulators. To do so, we designed a software adapter layer, which facilitates synchronized communication between Nengo and AirSim. The adapter aids in the reciprocal exchange of LiDAR readings and controls signals, which encompasses the values of a car's steering and throttle. We coordinated these scenarios to run concurrently with a minimal interval of 5 milliseconds, thereby imitating 200 control signals per second. This process effectively involves alternating between the two simulators every 5 milliseconds, during which one simulator operates while the other remains still. The Nengo simulator was calibrated with a physics delta time of 0.001 s to ensure an exceedingly accurate simulation. The intercommunication between Nengo and AirSim was made possible through the utilization of the AirSim API, which fundamentally operates on a TCP socket connection.

## 3. Results

This work's results span numerous neuromorphic and conventional models. The pure pursuit, Stanley, PID, and MPC controllers were implemented both neuromorphically using ensembles of spiking neurons and conventionally using a CPU. Each model's performance was evaluated using four matrices: the percentage of completed drives (getting from the startline to the finish regardless of collisions), the percentage of collision-free drives, the CTE, and average velocity. The performance of neuromorphic implementations was evaluated with varying numbers of neurons to provide insights regarding the required resources for adequate performance. The controllers' performances vary across different target velocities. We therefore further evaluated each model with different target speeds ranging from 2 to 20 m/s. The PID and MPC performances are also critically dependent on the value of the neuromorphic synaptic time constants (tau; the PID features three time constants and MPC has one). Therefore, the percentages of completed and collision-free drives were also evaluated with different time constants, and the results are given in the tables.

### 3.1. Pure-pursuit controller

**Figure 4:**
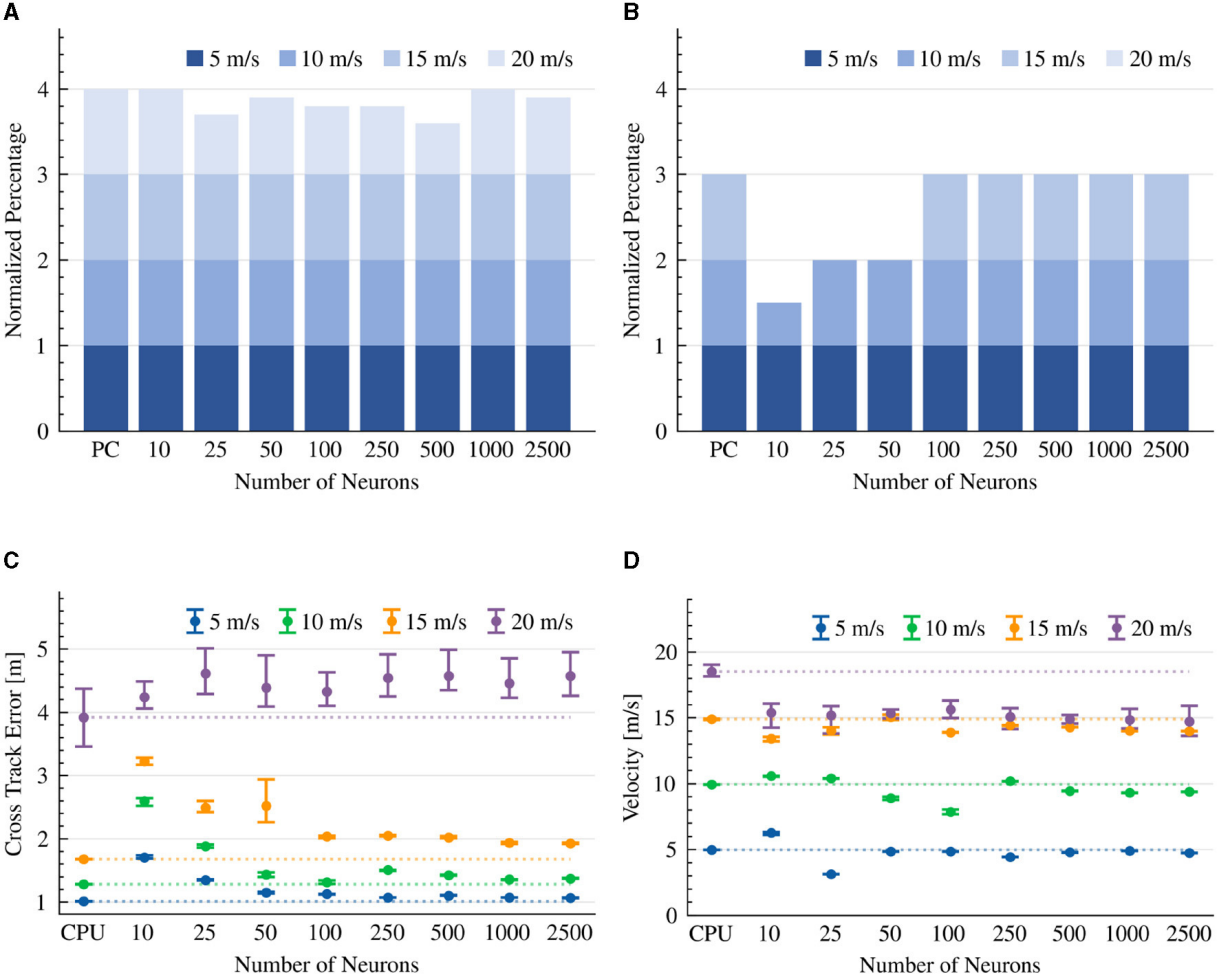
Pure-pursuit controller results. The CTE (RMS) and average velocity of experiments that were able to complete the lap. The dotted line is the average result of the CPU implementation as a reference line. **(A)** Completed drives. **(B)** Collision-free drives. **(C)** Cross track error (RMS). **(D)** Average velocity.

The results indicate that the CPU-based model completed the race course in all the evaluated scenarios. Our neuromorphic design was able to successfully complete a lap in all scenarios as well, with the exception of a few attempts to drive the vehicle at a high 20 m/s speed (Figure 4A). This result corresponds with the controller's reliance on ms-scale synaptic time constants. We note that at high speeds, both neuromorphic and CPU-based implementations hit the road's lining walls. The results indicate that with < 100 neurons, the neuromorphic-controlled vehicle touched the walls even at lower speeds, portraying the importance of neuronal resources for handling the car at high velocities (Figure 4B).

The results indicate that the RMS of the CTE is higher at high speeds, in both CPU and neuromorphic implementations. The CTE performance in the neuromorphic implementation converged at 100 neurons, where additional neurons did not improve its performance, suggesting a sweet spot for neuronal resources and efficiency. In lower velocities (5–10 m/s) and with sufficient >100 neurons, the neuromorphic model performed comparably well (0.12 to 0.23 meters) with the CPU-based implementation. At higher speeds (15–20 m/s), the CTE performance of the neuromorphic implementation increased up to 0.66 meters (Figure 4C). We show that with the exception of a high 20 m/s target speed, the CPU-based implementation was able to keep its target, while the neuromorphic design was less accurate (Figure 4D).

An important aspect of our neuromorphic design is the specified synaptic time constant (τ). We further tested the model's performance with 2 to 1,100 ms time constants, using 100 neurons and a 15 m/s target speed configuration. We show that when τ>10 ms the vehicle could not respond fast enough to successfully complete the race course (Table 1). The results indicate that the CPU-based implementation outperformed the neuromorphic version (time constant of 5 milliseconds, 15 m/s target speed), with a CTE of 1.68 m compared to 2.02 m (Table 1). In both CPU and neuromorphic implementations, the pure-pursuit model is steering aggressively at higher speeds, resulting in high CTE. However, at lower speeds, the neuromorphic implementation is comparable with the CPU implementation, while being powered by merely 100 spiking neurons.

**Table 1:** Results per different τ values for pure-pursuit neuromorphic implementation.

**τ [ms]**	**5**	**10**	**>10**
Completed Laps	100%	100%	0%
Collision-Free	100%	100%	0%
CTE [m]	2.02	2.03	–

### 3.2. Stanley controller

Like in the pure pursuit model, our neuromorphic design of the Stanley controller comprises a single neuron ensemble, which resolves Equation 13 and a dedicated neuromorphic PID controller that sets the vehicle's target speed. As a baseline, we set the output synaptic time constant to 10 ms.

**Figure 5:**
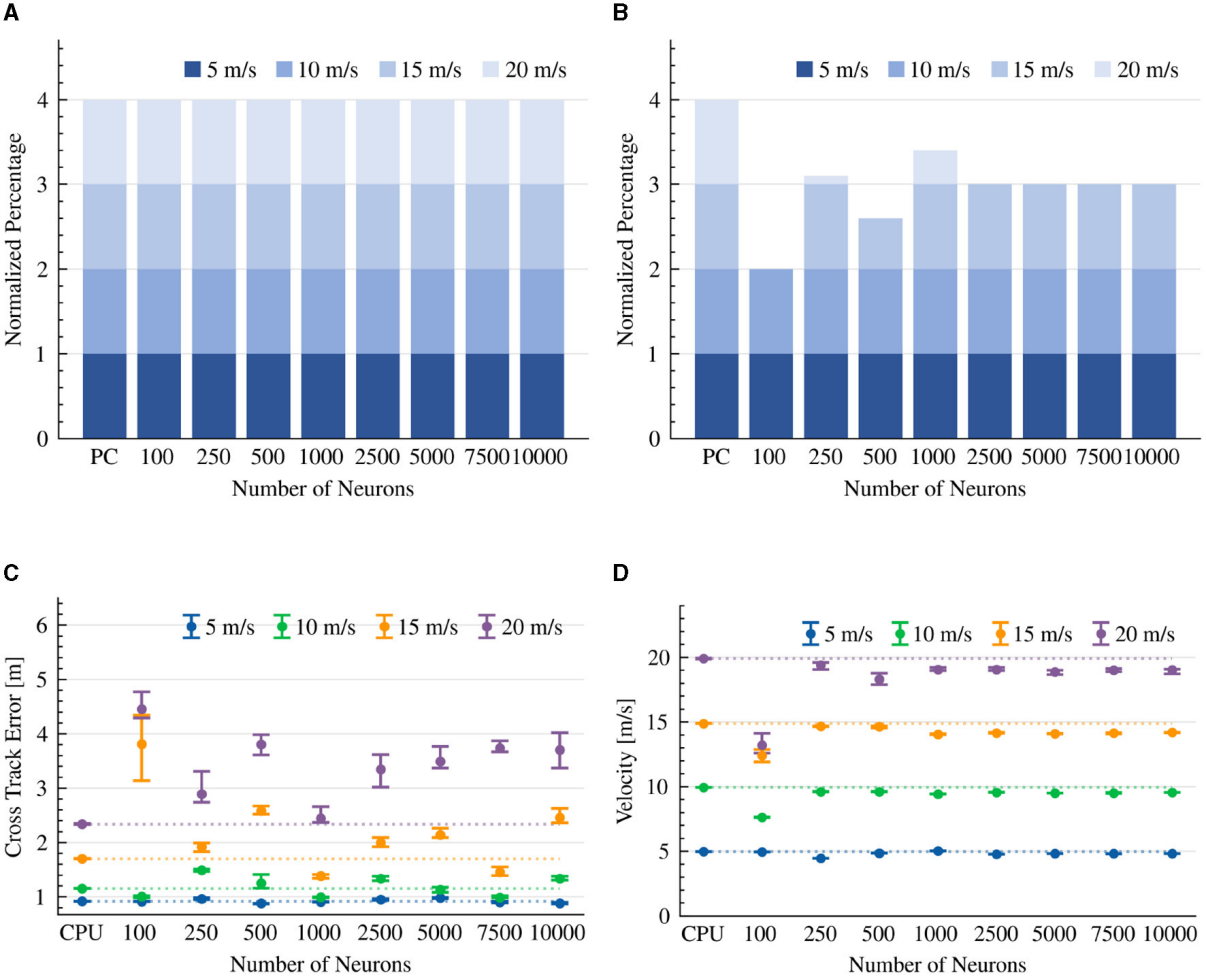
Stanley controller results. The CTE (RMS) and average velocity of experiments that were able to complete the lap. The dotted line is the average result of the CPU implementation as a reference line. **(A)** Completed drives. **(B)** Collision-free drives. **(C)** Cross Track Error (RMS). **(D)** Average velocity.

The results indicate that both the CPU-based implementation and our neuromorphic design were able to complete the race course in all of the tested scenarios (Figure 5A). However, while the CPU model completed each lap without collision with the road's boundaries, the neuromorphic implementation did touch the boundaries at 20 m/s in all experiments. However, it was able to drive without collision at 15 m/s when driven by >1,000 neurons (Figure 5B).

**Figure 6:**
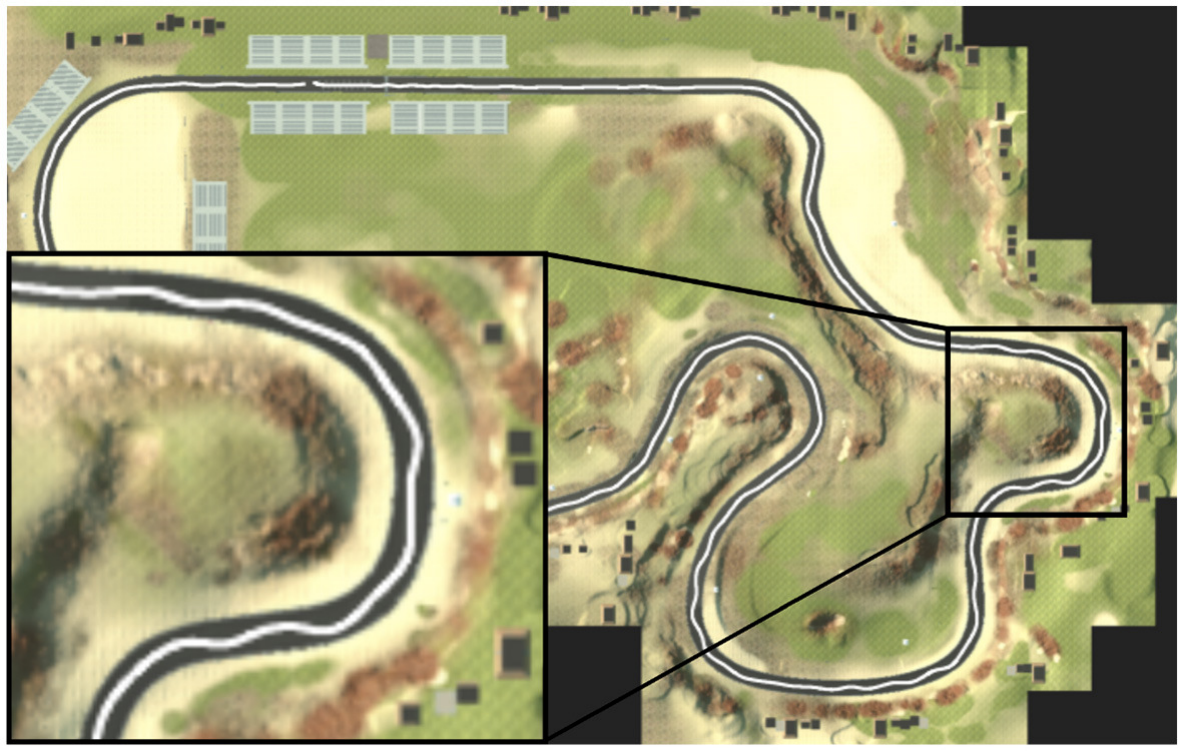
Zig-Zag patterns introduced in the neuromorphic Stanley controller with 2,500 neurons.

As expected, CTE performance was higher at higher speeds in both CPU and neuromorphic implementations. In a slow 5 m/s velocity, the 1000-neuron neuromorphic controller achieved a CTE of 0.90 m, comparable to the CPU performance of 0.92 m CTE on average. Surprisingly, as more neurons were allocated to the neuromorphic design, its performance shifted further away from the reference CPU's CTE (Figure 5C). However, looking carefully at the vehicle's diving path, we can see that with a low number of neurons, the car drove aggressively to keep the reference trajectory with sharp turns and Zig-Zag movement patterns. This is due to its insufficient capacity to accurately realize the Stanley driving equation (Equation 13). With 10,000 neurons, the vehicle driving path was much smoother, looking similar to the CPU implementation. However, we observed some drift from the reference trajectory. From the derived trajectories we conclude that with 2,500 neurons, the controller could drive the vehicle relatively smoothly with small Zig-Zag patterns (Figure 6).

Our results suggest that both CPU and neuromorphic-based implementations were generally able to maintain their target speed. However, our neuromorphic implementations were slightly less accurate. With >1,000 neurons, the vehicle was able to maintain speeds of 14 and 19 m/s on average, with 15 and 20 m/s target speeds, respectively (Figure 5D).

We further tested the effect of the synaptic time constant τ on the model's performance, evaluated on 1000 neurons and a 15 m/s target speed configuration. We show that in a neuromorphic controller with a > 50 ms τ, the controlled vehicle could not complete the race course successfully, indicating the importance of fast response time in neuromorphic systems. We show that while with a time constant of 50 ms, 90% of the drives were collision-free, with a time constant of ≤ 10 m, 100% of the drives were absent from collisions (Table 2). Overall, the performance of the neuromorphic Stanely controller was good but less accurate at 20 m/s, requiring at least 2,500 neurons to drive smoothly.

**Table 2:** Results per different τ values for Stanley neuromorphic implementation.

**τ [ms]**	**5**	**10**	**50**	**>50**
Completed Laps	100%	100%	100%	0%
Collision-Free	100%	100%	10%	0%
CTE [m]	1.72	1.39	3.23	–

### 3.3. PID controller

**Figure 7:**
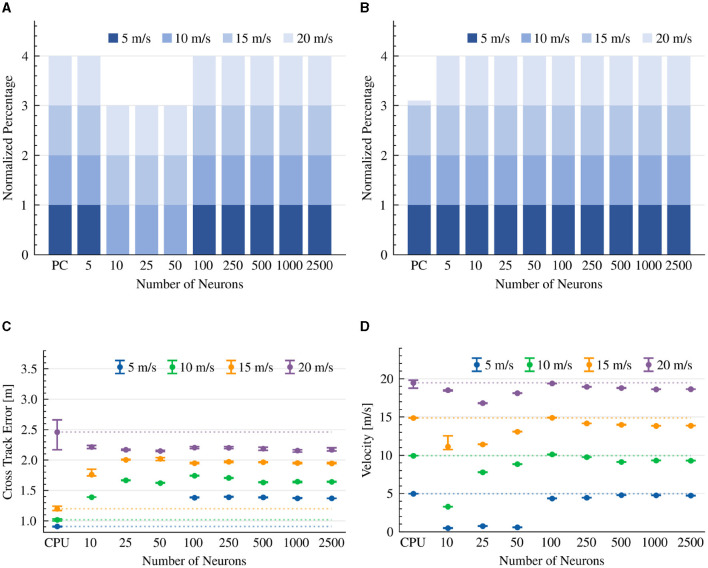
PID controller results. The CTE (RMS) and average velocity of experiments that were able to complete the lap. The dotted line is the average result of the CPU implementation as a reference line. **(A)** Completed drives. **(B)** Collision-free drives. **(C)** Cross Track Error (RMS). **(D)** Average velocity.

Our controller is comprised of two PID constructs, one responsible for steering and the other for speed. Our results show that with > 50 neurons per neuron ensemble, our neuromorphic PID controllers were sufficient to allow the vehicle to complete all drives with no collisions in the 5 to 20 m/s velocity range (Figure 7A). Our > 100 neurons configuration outperforms the CPU-based controller at a high 20 m/s velocity, perhaps suggesting a need to further optimize the prescribed PID gain coefficients (Figure 7B). With < 100 neurons our neuromorphic PID was not able to successfully complete a lap, even at a 5 m/s target speed. This is due to its limited capacity to represent small steering and throttle values to initiate movement (Figure 7D). We show that the CTE performance of the CPU-based PID implementation was high (with the exception of the 20 m/s target speed). The performance of the neuromorphic implementation plateaued with >100 neurons per ensemble, suggesting a more fundamental limit to the model's performance, probably the time-limiting synaptic constraints imposed on the computing of the PID's derivative and integral terms (Figure 7C). When considering target speed maintenance, the 500 neurons model was the closest to the 5 m/s target speed with a 4.8 m/s velocity, and the 100 neurons model was the closest to the 10, 15, and 20 m/s target speeds, with 10.12, 14.91, and 19.38 m/s velocities, respectively (Figure 7D).

We further tested our neuromorphic design with a 100 neurons steering PID controller and various synaptic time constants (for each of the proportional τ_*p*_, integral τ_*i*_, and derivative τ_*d*_ neural ensembles), at a 15 m/s target speed. Our results show that the lower these time constants get, the better the vehicle performance is, where with < 100 ms time constant, all drives were completed successfully. Results are summarized in Tables 3–5.

**Table 3:** Results per different τ_*p*_ values for the steering PID neuromorphic implementation.

**τ_*p*_ [ms]**	**5**	**10**	**50**	**100**	**300**
Completed Laps (rounded)	100%	100%	100%	100%	89%
Collision-Free (rounded)	59%	56%	68%	48%	34%
CTE [m]	2.66	2.62	2.54	2.65	3.18
τ_*p*_ **[ms]**	**500**	**700**	**900**	**1100**
Completed Laps (rounded)	89%	77%	69%	52%
Collision-Free (rounded)	25%	0%	0%	0%
CTE [m]	3.46	3.58	3.77	3.81

**Table 4:** Results per different τ_*i*_ values for the steering PID neuromorphic implementation.

**τ_*i*_ [ms]**	**5**	**10**	**50**	**100**	**300**
Completed Laps (rounded)	84%	80%	89%	85%	86%
Collision-Free (rounded)	36%	30%	30%	30%	36%
CTE [m]	2.98	2.98	3.11	3.05	3.06
τ_*i*_ **[ms]**	**500**	**700**	**900**	**1100**
Completed Laps (rounded)	85%	90%	88%	88%
Collision-Free (rounded)	36%	32%	33%	32%
CTE [m]	3.06	3.11	3.13	3.06

**Table 5:** Results per different τ_*d*_ values for the steering PID neuromorphic implementation.

**τ_*d*_ [ms]**	**5**	**10**	**50**	**100**	**300**
Completed Laps (rounded)	79%	77%	91%	100%	100%
Collision-Free (rounded)	0%	0%	0%	43%	69%
CTE [m]	3.97	3.91	3.64	2.86	2.46
τ_*d*_ **[ms]**	**500**	**700**	**900**	**1100**
Completed Laps (rounded)	100%	96%	88%	44%
Collision-Free (rounded)	63%	44%	35%	33%
CTE [m]	2.52	2.74	3.12	2.28

### 3.4. MPC Controller

**Figure 8:**
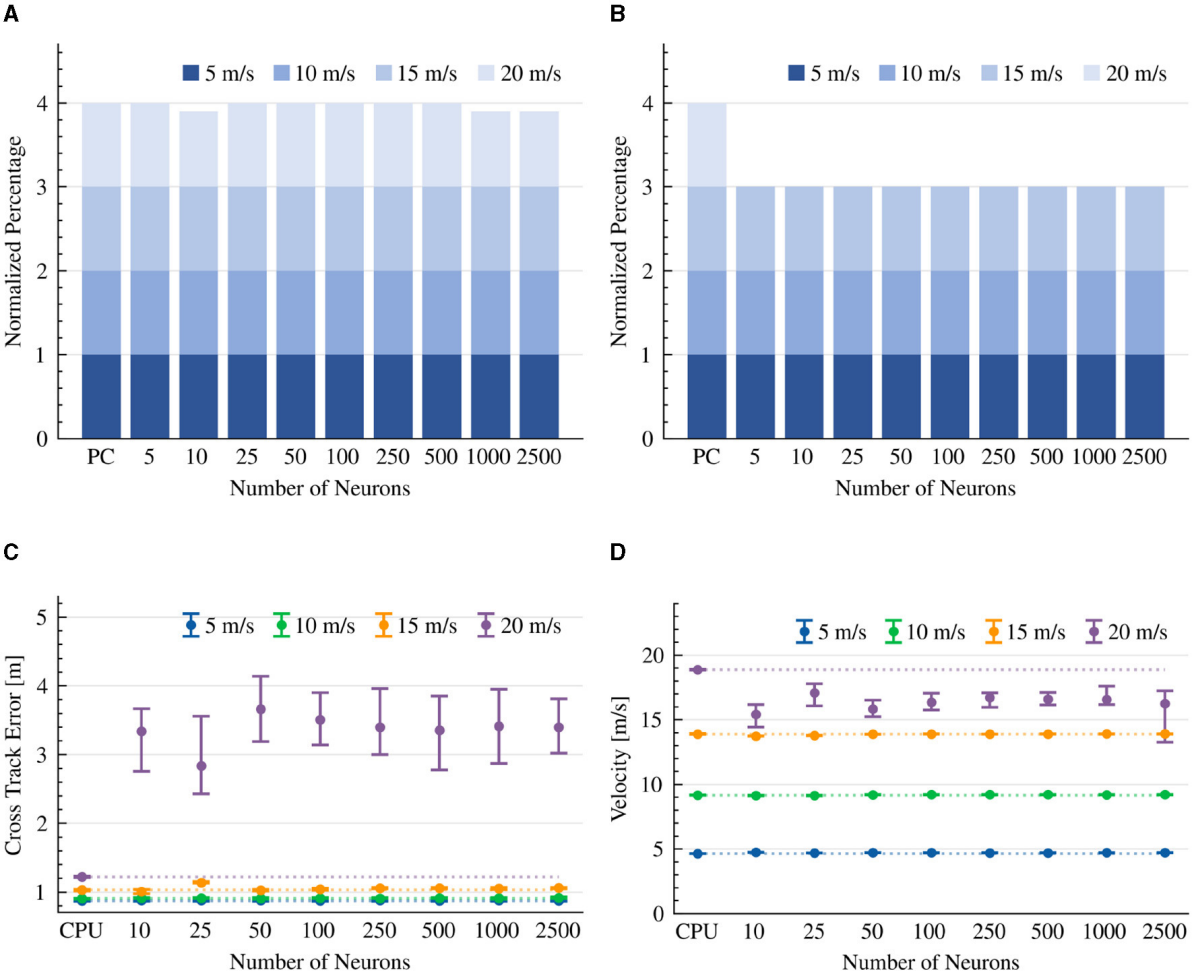
MPC controller results. The CTE (RMS) and average velocity of experiments that were able to complete the lap. The dotted line is the average result of the CPU implementation as a reference line. **(A)** Completed drives. **(B)** Collision-free drives. **(C)** Cross track error (RMS). **(D)** Average velocity.

To evaluate a neuromorphic MPC, we set the steering KBM-based MPC model to neuromorphically optimize the cost function (Equation 14), allowing simultaneous control of both steering and speed. Thus, in contrast to the previously implemented models, MPC controls the vehicle's velocity by considering several driving aspects. For example, the controller might reduce the vehicle velocity to allow for more accurate turns. With the exception of one drive, our neuromorphic MPC models were able to successfully complete all drives in the 5 to 20 m/s target velocity range (Figure 8A). However, at a high 20 m/s speed, the neuromorphic controllers failed to complete the race course without hitting the road's boundaries (Figure 8B). The CTE performance of the neuromorphic implementation was comparable with its CPU counterpart for all speeds with the exception of 20 m/s (Figure 8C). The neuromorphic and CPU-based implementations were able to maintain 4.63-4.74 m/s, 9-9.2 m/s, 13.69-13.91 m/s speeds on average, at target speeds of 5, 10, and 15 m/s, respectively. However, at 20 m/s, the CPU was able to maintain 18.87 m/s on average, while the neuromorphic implementation was able to only maintain a speed of 15.54-17.17 m/s on average (Figure 8D).

We further tested the effect of the synaptic time constants on the neuromorphic model performance using a 100-neuron, 15 m/s configuration. With > 300 ms time constants, our neuromorphic model could not complete the race course successfully. With time constants of 10, 50, and 100 ms we measured the lowest CTE values of 1.04. Results are summarized in Table 6.

**Table 6:** Results per different τ_*p*_ values for steering MPC neuromorphic implementation.

**τ [ms]**	**5**	**10**	**50**	**100**	**300**	**>300**
Completed Laps	100%	100%	100%	100%	100%	0%
Collision-Free	100%	100%	100%	100%	100%	0%
CTE [m]	1.06	1.04	1.04	1.04	1.10	–

Uniquely, we were able to implement an MPC-hybrid model by realizing the mathematical computation on a CPU and using the neuromorphic model as the optimizer that chooses the policy. This hybrid model shows how the advantages of two processing units could be integrated: a regular CPU for accurate numerical calculations and a neuromorphic processor for low power and continuous optimization, without an internal clock. We show that the MPC-hybrid implementation performs well even with 10 neurons per ensemble (with the exception of a high 20 m/s target speed).

## 4. Discussion

The results of our experiments demonstrate the potential of neuromorphic control in the context of autonomous driving. We showed that the neuromorphic implementations of the Pure-pursuit, Stanley, PID, and MPC controllers were able to perform competitively with their CPU-based counterparts, particularly at lower speeds. This highlights the viability of using neuromorphic control systems for autonomous driving applications, offering energy-efficient alternatives to traditional methods.

One key finding from our experiments is the importance of tuning parameters, such as the synaptic time constant and the number of neurons to achieve optimal performance, suggesting a sweet spot for neuronal resources and efficiency. For example, our results show that while the neuromorphic Pure-pursuit controller's performance converges at 100 neurons, Stanley controller requires more than 1000 neurons to converge. PID and MPC controllers require lower neuronal resources (10 neurons per ensemble) but are prone to time-constant adjustments. Additionally, the results indicate that the choice of synaptic time constant can significantly impact the controller's ability to respond quickly and accurately to environmental changes. All neuromorphic models performed well within the 0 to 15 m/s target velocity range, wherein higher velocities their performances degrade. Another important aspect of our research is the use of hybrid neuromorphic-CPU controllers, such as the MPC implementation. These hybrid controllers leverage the strengths of both neuromorphic and traditional computing systems, providing a promising avenue for future research in the field of autonomous driving.

Despite the promising results, there are several areas that warrant further investigation. Our study highlights the limitations of neuromorphic implementations, particularly at higher speeds. With high target velocities, the performance of the neuromorphic controllers tends to degrade faster than the CPU implementation, with higher cross-track errors and less accurate velocity control. This result is not surprising when rate-coded neuromorphic representation is used. Therefore, architectural insights from conventional neural circuits (Tian et al., 2023) or more advanced neuromorphic designs, which incorporate other representation modalities, such as time to spike, should be explored. The incorporation of further feedback from visual sensing (Shi et al., 2023b) and other sensing modalities (Shi et al., 2023a) could be used to improve the car's ability to adaptively respond the environmental changes.

The derivation of real-life vehicle performance might highlight additional concerns, such as hardware compatibility and safety concerns. Furthermore, the performance of the models on real neuromorphic hardware and physical cars could assess their scalability and power performance. Mainly, hardware deployment is highly dependent on software-hardware compliance. The neuronal architecture could be further optimized for the specific hardware optimization, which was recently demonstrated using various learning strategies (Liu et al., 2023b; Wang et al., 2023).

In conclusion, our study provides insights into the potential of neuromorphic control for autonomous driving applications. The competitive performance of the neuromorphic implementations compared to their CPU-based counterparts demonstrates the promise of this approach. Future research should focus on addressing the limitations of neuromorphic controllers at high speeds and exploring the potential of hybrid neuromorphic-CPU systems for improved performance and energy efficiency.

## Data availability statement

The original contributions presented in the study are included in the article/supplementary material, further inquiries can be directed to the corresponding author.

## Author contributions

All authors listed have made a substantial, direct, and intellectual contribution to the work and approved it for publication.
